# American trypanosomiasis and associated risk factors in owned dogs from the major city of Yucatan, Mexico

**DOI:** 10.1186/s40409-015-0039-2

**Published:** 2015-09-30

**Authors:** Matilde Jiménez-Coello, Karla Acosta-Viana, Eugenia Guzmán-Marín, Alejandra Bárcenas-Irabién, Antonio Ortega-Pacheco

**Affiliations:** Laboratorio de Biología Celular, Centro de Investigaciones Regionales “Dr. Hideyo Noguchi”, Universidad Autónoma de Yucatán, Mérida, Yucatán Mexico; Unidad de Enseñanza Médica, Campus de Ciencias Biológicas y Agropecuarias, Universidad Autónoma de Yucatán, km 15.5 Carretera Mérida-Xmatkuil, AP 4-116 Mérida, Yucatán Mexico

**Keywords:** *Trypanosoma cruzi*, Epidemiology, Owned dogs, Major city, Mexico

## Abstract

**Background:**

The American trypanosomiasis is a zoonosis caused by the protozoa *Trypanosoma cruzi* (*T. cruzi*). The disease is widely distributed throughout the American continent, affecting a wide range of hosts, including dogs. It is present in the canine population in the State of Yucatan, Mexico. However, no significant studies in owned dogs have been performed in the metropolitan area of Merida. A transversal study was conducted in 370 owned dogs from Merida, Yucatan, Mexico.

**Methods:**

A cross-sectional study including 370 dogs was performed in a major city of Yucatan, Mexico, to detect IgG antibodies against *T. cruzi*. A commercial ELISA test kit was used and a chi-square test used to evaluate associated risk factors; odds ratio (OR) and 95 % confidence interval (CI) were also estimated.

**Results:**

The indirect ELISA and western blot (WB) tests were used to detect specific immunoglobulin G antibodies against *T. cruzi* in serum samples. A prevalence of 12.2 % was found; age and area of residence were statistically associated with seropositivity in dogs (*p* <0.05).

**Conclusions:**

Results from the present study suggests the presence and abundance of the vector in urban conditions where a high number of seropositive cases of *T. cruzi* cases were found.

## Background

American trypanosomiasis, or Chagas disease, is a zoonosis of public health concern caused by the protozoon *Trypanosoma cruzi* and transmitted by a bloodsucking bug from the Triatominae subfamily. In Yucatan, Mexico, the disease is endemic and affects a wide range of hosts, including dogs and humans [1, 2]. Triatominaes may prefer to feed on dogs, which then become reservoirs of the agent and become involved in the intra-domiciliary transmission cycle [3–6]. Once infected, dogs may develop clinical signs of the disease, which are mainly characterized by cardiac insufficiency. If the dog survives, it can become chronically infected [7]. Traditionally, rural areas are considered to be at higher risk for infection through the vector’s bite, but this risk is also present in stray dogs from urban areas [1, 8]. Owned dogs are in close contact with their owners, and thus, they are at a higher risk of transmitting diseases, including Chagas [9, 10]. A preliminary study in the city of Merida indicated that 34 % of owned dogs and 8 % of their owners were infected with American trypanosomiasis [2].

The objectives of this study were to determine the seroprevalence of American trypanosomiasis in owned dogs from the city of Merida, Yucatan, Mexico, and to determine the associated risk factors.

## Methods

### Study area

The study was conducted in the city of Merida, located northwest of the state of Yucatan, Mexico (19° 30′ and 21° 35′ north latitude, 87° 30′ and 90° 24′ west longitude). The city’s climate is warm and sub-humid with summer rains and is 6 m above sea level [11].

### Sampling

A total of 370 owned dogs from the metropolitan area of Merida were included. Sample size was determined considering a population of at least 200,000 owned dog in the city with an expected prevalence of 17 %, with a 99 % of confidence level and 5 % of precision [1, 12]. Blood samples were collected from the cephalic vein during a spaying campaign. Serum was obtained by centrifugation of the samples at 2700 *g* for 5 min. During the sample collection, owners were asked their home address and data about their dogs (age and sex). Blood samples from dogs were collected with the consent of their owners after explaining the objectives of the study. The study was approved by the Bioethics Committee of the Campus de Ciencias Biológicas y Agropecuarias, Universidad Autónoma de Yucatán (CB-CCBA I-2014-003).

### Serological detection

The immunoglobulin G (IgG) indirect ELISA test was performed as previously described using the commercial ELISA test kit (Chagatest ELISA recombinant v.3.0, Wiener, Argentina) [1, 8]. Cultured parasites of the reference *T. cruzi* Y strain were used as antigens and the methodology was previously described [1]. To confirm the serologic diagnosis, the western blot (WB) method was used according to a methodology previously described by Teixeira *et al.* [13].

### Statistical analysis

The study was designed as a cross-sectional study, and the results were analyzed using descriptive statistics to determine the prevalence of *T. cruzi*. A case was considered true positive when it was positive for two serological tests (ELISA and WB). To determine the association among the studied variables (age, gender and area of residence of the dogs) and the presence of specific antibodies to *T. cruzi*, a chi-square test was used; the odds ratio (OR) and 95 % confidence interval (CI) were also estimated. SPSS v.15.0 was used with a significance level of *p* <0.05.

The area of residence was divided into four zones considering their socioeconomical status previously reported, the building characteristics of households and closeness to forestry areas [12]. Southeast and southwest zones are of lower socioeconomical status with several houses of straw roof and earthen floor at the periphery of the city and with abundant natural forestry.

## Results and discussion

**Fig. 1:**
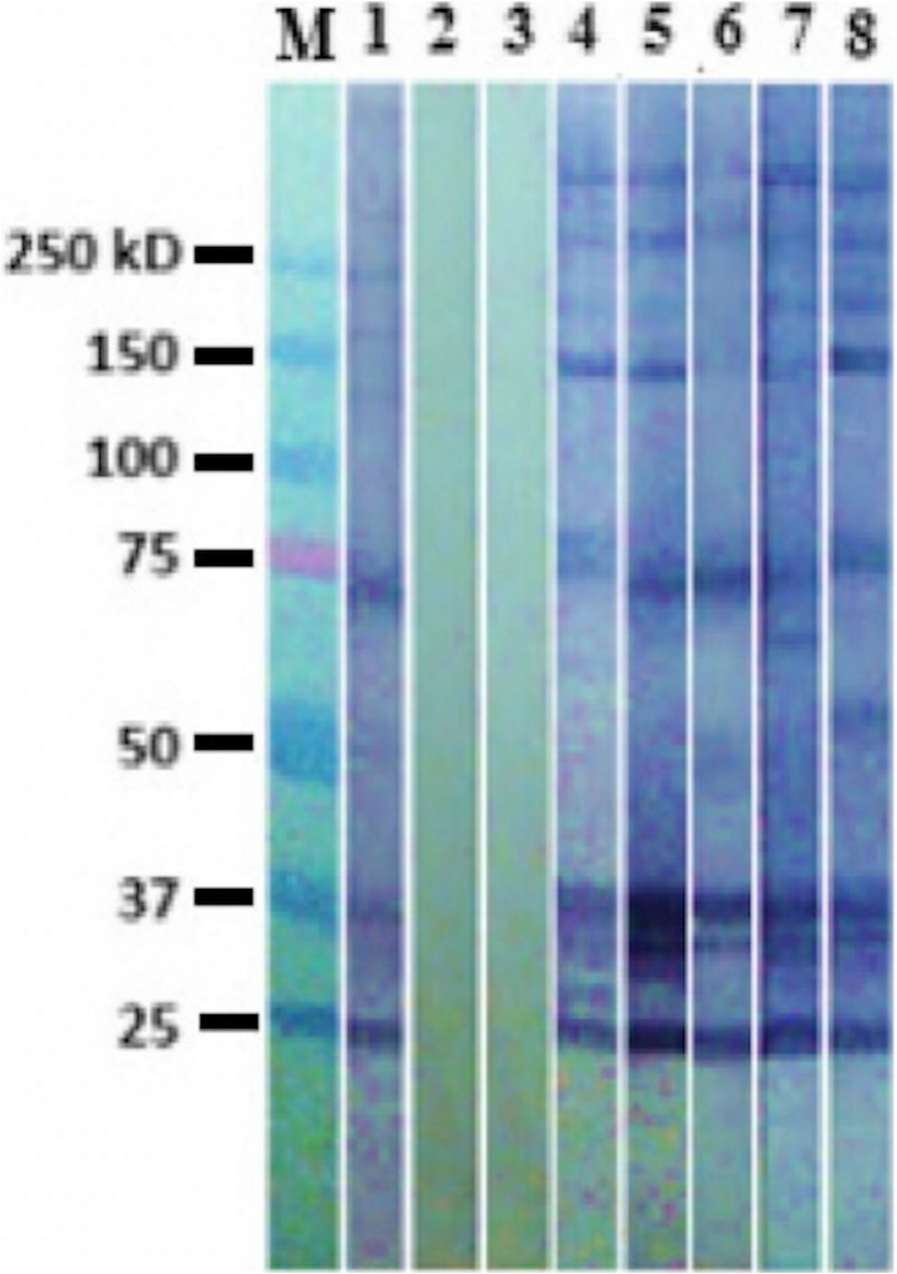
Example of western blot to confirm the presence of IgG antibodies against *T. cruzi* in dogs positive to indirect ELISA. Lane M: molecular weight marker Biorad cat. 161–0373 All Blue 250 kD; lane 1: positive control (serum from a dog confirmed as positive); lane 2: negative control (serum from a dog confirmed as negative); lane 3; control of dog anti-IgG; lanes 4–8: samples of owned dogs in the metropolitan area of Merida, Yucatan, reactive to *T. cruzi*

Among the 370 samples, seroprevalence was 12.2 % (95 % CI: 9.5–15.1 %). An example of the western blot is shown in Fig. 1. Regarding the risk factors considered, only the area of the residence (southeast) had a significant effect on the seropositivity of the dogs. Dogs older than two years had a 1.85-fold greater risk (*p* >0.06) of becoming infected with *T. cruzi* compared to younger dogs (Table 1).

**Table 1:** Risk factors, odds ratios and confidence intervals associated with positive results for *T. cruzi* in 370 owned dogs of Merida, Yucatan

Risk factor	n	Positive	Frequency (%)	OR	CI 95 %	*p* value
Sex						
Female	278	34	12.23^a^	1.03	0.47-2.26	0.94
Male	92	11	11.95^a^	1		
Age						
≥2	208	31	14.9^a^	1.85	0.9-3.81	0.06
<2	162	14	8.64^a^	1		
Domiciliary area						
A (northwest)	118	12	10.16^a^	0.45	0.19-1.06	
B (northeast)	97	8	8.2^a^	0.38	0.15-0.98	
C (southwest)	85	11	12.94^ab^	0.69	0.28-1.68	
D (southeast)	70	14	20^b^	1		0.05
Total	370	45	12.16			

*OR* Odds ratio, *CI* Confidence interval

Different letters (a, b) indicate statistical significance (*p* <0.05)

In addition, the role of owned dogs (domiciliary dogs) as reservoirs of *T. cruzi* in the city of Merida was studied. Although the prevalence of *T. cruzi* has been widely reported in urban dogs, most of the studies were based on stray dogs from cities or villages, or a low number of owned dogs were included. A survey conducted on 148 stray dogs and 114 pet dogs in a similar city from the Yucatan Peninsula (Campeche) reports *T. cruzi* seroprevalences of 9.5 and 5.3 %, respectively [14]. In Costa Rica, the prevalence in pet dogs varies from 5.2 % (*n* = 58) to 1.6 % (*n* = 51) from endemic and non-endemic areas, respectively [15]. Several studies in Texas, United States, demonstrate the presence of *T. cruzi* in owned dogs with frequencies ranging from 12 % (*n* = 136 dogs) to 15 % (*n* = 356 dogs) [16, 17]. Other USA states report seroprevalences of 4 % (*n* = 301 dogs) in Oklahoma, 22 % (*n* = 50 dogs) in Louisiana and 6 % (*n* = 860 dogs) in Tennessee [18–20]. The exposure of pet dogs to the infected vector demonstrates the risk to humans living in the same households [9, 21, 22]. The variation in the prevalence in different studies may be due to the presence and abundance of the vector in each studied region; the presence of vectors depends on climatic, environmental and geographical conditions that are ideal for their survival and reproduction. The presence of the vector may be closely associated with different social and economic conditions of each region; the survival and reproduction of vectors may favored in low economic conditions [5, 14, 23].

These results indicate that the vector adapts quickly to houses, gardens and parks in the city and can spread quickly when urbanization is introduced into rural areas. The blood of dogs may be an important source of triatomine bugs [24]. The vector *T. dimidiata* is well established within the municipality of Merida, Yucatan [25]. Dogs can act as sentinels of domestic and peridomestic vector-mediated transmission of *T. cruzi*, which was demonstrated in the Chaco province in Argentina and can be used as a control group if canine infections acquired by all other routes could be excluded [4]. Results from the present study suggest that dogs from the south area of the city are at higher risk of infection. In this particular area, the socioeconomic deprivation index is higher compared to the northern area [26]. The higher frequency of infected dogs in the south may be due to the characteristics of the construction of houses: for example, when roofing is made of aluminum sheets or guano or when cracks are present in the in walls where vectors can nest [27]. Furthermore, urbanization and the rapid growth of cities into rural areas increased the risk of vector transmission to dogs and humans because of their invasion into the natural areas of the vectors. As reported in other urban cities, trypanosomatids are increasingly closer to urban centers and highly associated with human cases [9]. In the present study, despite age not being a significant factor (*p* <0.06), older dogs are usually at a higher risk of being in contact with the vector; this result is similar to a result previously reported [1, 2].

## Conclusions

The American trypanosomiasis is present in a large proportion of dogs living in the city of Merida. This result is most likely due to the ability of vectors to adapt to the urban conditions, particularly the ideal conditions for vector nesting and multiplication found in the south of the city. Therefore, a surveillance program should be implemented and inhabitants must be educated about the risks for infection and practice vigilance in order to lower these risks for pets and humans.

### Ethics committee approval

The present study was approved by the Bioethics Committee of the Campus de Ciencias Biológicas y Agropecuarias from the Universidad Autónoma de Yucatán (CB-CCBA I-2014-003). In addition, dog blood samples were collected with their owners’ consent after explaining the objectives of the study.
